# Performance Study of MXene/Carbon Nanotube Composites for Current Collector‐ and Binder‐Free Mg–S Batteries

**DOI:** 10.1002/cssc.202100173

**Published:** 2021-02-24

**Authors:** Henning Kaland, Frode Håskjold Fagerli, Jacob Hadler‐Jacobsen, Zhirong Zhao‐Karger, Maximilian Fichtner, Kjell Wiik, Nils P. Wagner

**Affiliations:** ^1^ Department of Materials Science and Engineering NTNU Norwegian University of Science and Technology 7491 Trondheim Norway; ^2^ Helmholtz Institute Ulm (HIU) Electrochemical Energy Storage Helmholtzstr. 11 89081 Ulm Germany; ^3^ Institute of Nanotechnology Karlsruhe Institute of Technology (KIT) P.O. Box 3640 76021 Karlsruhe Germany; ^4^ SINTEF Industry Sustainable Energy Technology 7465 Trondheim Norway

**Keywords:** batteries, magnesium, MXenes, nanotubes, sulfur

## Abstract

The realization of sustainable and cheap Mg‐S batteries depends on significant improvements in cycling stability. Building on the immense research on cathode optimization from Li‐S batteries, for the first time a beneficial role of MXenes for Mg‐S batteries is reported. Through a facile, low‐temperature vacuum‐filtration technique, several novel current collector‐ and binder‐free cathode films were developed, with either dipenthamethylene thiuram tetrasulfide (PMTT) or S_8_ nanoparticles as the source of redox‐active sulfur. The importance of combining MXene with a high surface area co‐host material, such as carbon nanotubes, was demonstrated. A positive effect of MXenes on the average voltage and reduced self‐discharge was also discovered. Ascribed to the rich polar surface chemistry of Ti_3_C_2_T_*x*_ MXene, an almost doubling of the discharge capacity (530 vs. 290 mA h g^−1^) was achieved by using MXene as a polysulfide‐confining interlayer, obtaining a capacity retention of 83 % after 25 cycles.

## Graphical abstract

**Layer by layer**: A positive effect of MXenes on capacity retention and reduced self‐discharge for Mg‐S batteries is demonstrated, either as a cathode constituent or as an interlayer between the cathode and the separator. An almost doubling of the discharge capacity (530 vs. 290 mA h g^−1^) is achieved by using MXene as a polysulfide‐confining interlayer, obtaining a capacity retention of 83 % after 25 cycles.

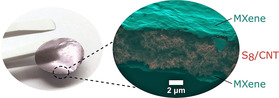

## Introduction

Mg‐S batteries represent an outstanding candidate for cheap, sustainable, energy‐dense, and safe energy storage, which is of utmost importance for accelerating the transition to a fully renewable energy‐based society. In contrast to state‐of‐the‐art Li‐ion batteries that typically comprise Ni and Co, Mg‐S batteries rely on abundant, non‐toxic Mg and S as electrochemically active materials. The risk of uneven Li deposition during charging (so‐called dendrites) causing internal short circuiting remains a safety concern for conventional graphite‐based Li‐ion insertion anodes,^[1]^ and the risk is even more prominent for the long‐wanted Li metal anodes.^[2]^ Albeit not being dendrite‐immune,^[3]^ Mg metal anodes have an inherently lower propensity for dendrite formation than Li metal, possibly due to faster self‐diffusion.[^4^, ^5^] This could enable safe operation of Mg metal anodes, allowing a huge anode specific capacity of 2205 mA h g^−1^ and volumetric capacity of 3833 mA h cm^−3^. Combined with the high capacity of sulfur (1672 mA h g^−1^ and 3461 mA h cm^−3^) with a theoretical voltage of 1.77 V (assuming rock salt MgS as end product),^[6]^ the theoretical specific energy calculates to approximately 1700 W h kg^−1^ and the volumetric energy density to approximately 3200 W h L^−1^ for a Mg‐S full cell at the material level with 0 % Mg excess. With the experimentally observed zinc blende MgS as the end product, an average voltage of 1.4 V may be more accurate,^[7]^ yet this still calculates to a high value of approximately 1300 W h kg^−1^ and 2500 W h L^−1^.

Despite its great promise, the progress of Mg‐S batteries has been hampered by several non‐trivial challenges. Identifying high‐performing electrolytes compatible with the reductive nature of Mg metal has itself been a challenge from the first investigation of non‐aqueous Mg batteries in 1990.^[8]^ With the additional constraint that the electrolyte must be non‐nucleophilic due to the electrophilic nature of sulfur,[^9^, ^10^, ^11^] the first demonstration of a reversible Mg‐S battery was not realized before 2011 by Kim et al.^[9]^ Supported by X‐ray photoelectron spectroscopy (XPS), they proposed that S forms MgS through soluble polysulfides (MgS_*x*_, 1<*x*<8).^[9]^ Some of the intermediate soluble polysulfides may diffuse to the anode and be reduced, causing overcharge and capacity fading,[^9^, ^11^, ^12^] which is analogous to the heavily researched Li‐S batteries.^[13]^ This so‐called polysulfide shuttling is regarded as one of the remaining critical challenges for both Mg‐S and Li‐S batteries.[^11^, ^12^, ^13^] The reaction mechanism has largely been verified by later studies, yet the exact reaction steps and sulfide species may depend on the electrolyte and current density.[^7^, ^14^, ^15^, ^16^, ^17^]

Significant improvements have been reported for non‐nucleophilic electrolytes for Mg‐S batteries since 2011.[^10^, ^14^, ^18^, ^19^, ^20^, ^21^] It is worth to mention that the practicability of an electrolyte is of crucial importance for the realization of Mg‐S batteries. Some chlorine‐containing electrolytes such as Mg(HMDS)_2_‐AlCl_3_ [HMDS=hexamethyldisilazide] and Mg(TFSI)_2_‐MgCl_2_ [TFSI=bis(trifluoromethane)sulfonimide] have been initially investigated for Mg–S batteries.[^10^, ^14^] However, the corrosive nature of the chloride ion restricts their practical applications. Recently, the accomplishment in chlorine‐free electrolytes based on the conductive ionic compound magnesium tetrakis(hexafluoroisopropyloxy) borate, Mg[B(hfip)_4_]_2_ [hfip=OC(H)(CF_3_)_2_], has provided new prospects for Mg‐S batteries.[^20^, ^22^]

Contrary to the significant research efforts on non‐nucleophilic electrolytes, not much attention has been dedicated to cathode optimization.^[23]^ This is in stark contrast to Li‐S batteries, where the cathode has been the most studied cell component.^[13]^ Due to sulfur being ionically and electronically insulating,[^24^, ^25^] a high‐surface‐area material with high electronic conductivity is typically combined with sulfur in the cathode, where different conductive allotropes of carbon have been commonly used.[^13^, ^26^] However, despite being low‐cost and light‐weight, the non‐polar carbonaceous surface only interacts with the polar polysulfides through physisorption, and it is widely recognised that a polar surface obtained via, for example, doping or metal‐non‐metal bonds can confine polysulfides stronger through chemical adsorption and thus effectively reduce the polysulfide shuttling.[^13^, ^26^] As a result, a myriad of sulfur cathode architectures have been investigated for Li‐S batteries, ranging from simple elemental blends to doped hierarchical core‐shell porous structures comprising graphitic carbon, graphene, carbon nanotubes (CNTs), carbon nanofiber, metal oxides, metal sulfides, metal hydroxides, metal‐organic frameworks (MOFs), conductive polymers, organosulfur compounds, and/or MXenes.^[13]^ With a few exceptions of nitrogen‐doped hybrid nanocarbon^[7]^ and MOF‐derived carbon,^[27]^ relatively simple carbon‐sulfur cathodes have predominantly been reported for Mg‐S batteries thus far.^[23]^ Hence, there is an opportunity to improve the cyclability of Mg‐S batteries by taking advantage of the vast cathode research from Li‐S batteries.

One highly interesting candidate as sulfur host material is the broad group of 2D transition metal carbides, nitrides, and carbonitrides called MXenes,^[25]^ first discovered in 2011.^[28]^ They are commonly described by the formula M_*n*+1_X_*n*_T_*x*_, where M represents an early transition metal (i. e., Ti, V, Nb, Mo), X represents C and/or N, and T_*x*_ represents surface termination groups of −O, −F, −OH, and/or −Cl.^[29]^ First reported by Liang et al. as a sulfur host material for Li–S batteries in 2015,^[30]^ MXenes have in recent years been demonstrated to significantly increase sulfur utilization and capacity retention in numerous MXene‐based cathodes for Li‐S batteries.^[25]^ The improved electrochemical performance has been mostly ascribed to the MXenes’ high electronic conductivity and rich surface chemistry.^[25]^ Intriguingly, not only does the polar surface chemistry offer polar‐polar interactions with the polar polysulfides, but also Lewis acid–base chemisorption.[^25^, ^26^] The latter involves the formation of a metal‐sulfur bond and represents the strongest type of polysulfide bonding.^[26]^ In contrast to, for example, MOFs and metal hydroxides that also offer Lewis acid‐base chemisorption but have low electronic conductivity,^[13]^ the most common MXene, Ti_3_C_2_T_*x*_, has demonstrated one of the highest electrical conductivities of all solution‐processed nanomaterials.^[29]^ Moreover, the MXene −OH termination groups have been shown to form thiosulfates and electrochemically active^[26]^ polythionates by reacting with polysulfides,^[31]^ which represents a third important polysulfide confinement mechanism.^[26]^ Adding hydrophilicity, excellent mechanical properties, and structural flexibility,^[29]^ it is not surprising MXenes have been intensively reported both as a cathode constituent[^30^, ^31^, ^32^, ^33^, ^34^, ^35^, ^36^, ^37^, ^38^, ^39^, ^40^, ^41^, ^42^, ^43^, ^44^, ^45^, ^46^, ^47^, ^48^, ^49^, ^50^, ^51^, ^52^] and as an interlayer between the cathode and the separator[^33^, ^36^, ^43^, ^48^, ^53^, ^54^, ^55^, ^56^, ^57^, ^58^, ^59^] for Li‐S batteries. The main disadvantages of MXenes are the typical preparation route via toxic HF etching and that they are prone to restack, causing a low surface area.^[13]^ To address the latter, hybridizing MXene with a high‐surface‐area co‐matrix material^[25]^ that can have the synergetic effect of preventing the MXene restacking[^31^, ^60^] stands out as a highly promising route.

In this work, we are the first to report MXenes as a sulfur host material for Mg‐S batteries. We investigate several new easily synthesized MXene‐based cathode architectures aimed at increasing the sulfur utilization and cycle life while reducing the amount of inactive weight. First, we use a recently studied non‐toxic organosulfur compound from the rubber industry, dipenthamethylene thiuram tetrasulfide (PMTT),^[61]^ as the source of redox active sulfur, and prepare current collector‐ and binder‐free MXene‐based composites through a low‐temperature wet‐chemical procedure. With the non‐corrosive, non‐nucleophilic electrolyte based on the Mg[B(hfip)_4_]_2_ salt,^[20]^ the synergistic effect of MXenes and CNTs are evaluated. Then, we extend and further develop the procedure to S_8_ nanoparticles as active material and report the first use of MXene films as highly effective interlayers for Mg‐S batteries.

## Experimental Section

### Synthetic procedures

Synthesis of delaminated Ti_3_C_2_T_*x*_ MXene: An aqueous dispersion of delaminated Ti_3_C_2_T_*x*_ was obtained based on the optimized MILD method.^[62]^ In short, 0.8 g LiF (Sigma Aldrich, 99.995 %) was dissolved in 10 mL 9 m HCl (Sigma Aldrich, diluted from 37 % ACS reagent grade) to form in situ HF by 5 min stirring with a Teflon‐coated magnet. Then, 0.5 g Ti_3_AlC_2_ (Laizhou Kai Kai Ceramic Materials Co., Ltd, >98 %, 200 mesh) was added over the course of 5–10 min due to the exothermic reaction. A parafilm with a small hole was used as a lid to retard evaporation and simultaneously prevent hydrogen gas accumulation. After 24 h, the mixture was washed several times with deionized H_2_O by centrifugation (4350 rpm, ≈1800 g for 7 min using a VWR Mega Star 600 and 125 mL plastic bottles). After each washing cycle, the transparent supernatant was decantated and the wet powders carefully redispersed by light shaking. Typically, after 3–4 washing cycles, the dark supernatant did not sediment after the centrifugation, indicating that the delamination of MXene had started. At this point, the bottle was thoroughly hand‐shaken for 5 min, followed by centrifugation at 3500 rpm for 7 min. Lastly, the stable dispersion of delaminated MXene was collected as the supernatant. The concentration of the dispersion was found by vacuum filtering a certain amount of the dispersion and weighing the resulting dried MXene. Two different MXene batches were used in this work, and the concentration was controlled at 0.25 g L^−1^ by dilution with deionized H_2_O. Upon storage, the dispersion was deoxygenated with argon gas, sealed, and stored in a fridge to prevent oxidation.^[63]^

**Scheme 1:**
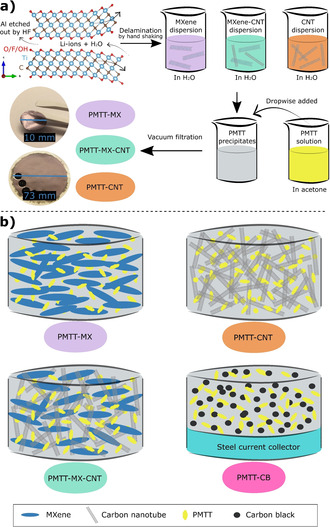
(a) Procedure for preparing PMTT composites from Ti_3_C_2_T_*x*_ MXene, CNT, and PMTT, and (b) schematic of the resulting films. A schematic of a conventionally fabricated electrode by casting on stainless‐steel current collector with carbon black as conductive additive (PMTT‐CB) is also included.

Preparation of PMTT composites: The current collector‐ and binder‐free electrodes were prepared through a vacuum‐assisted filtration technique (illustrated in Scheme 1). First, PMTT (Tokyo Chemical Industry, 58 % sulfur) was dissolved in acetone (0.0125 g in 6.3 mL acetone) at 70 °C on a hot plate for 5–10 min. To prepare the 50 : 50 wt % PMTT‐MXene composite (abbreviated PMTT‐MX), the PMTT solution was added dropwise to 50 mL of the 0.25 g L^−1^ delaminated Ti_3_C_2_T_*x*_ aqueous dispersion under strong stirring. After stirring for 10 min, the resulting mixture was vacuum filtered onto Celgard 3501 membrane paper (64 nm pore size). A fritted glass vacuum filtration equipment (Whatman™ 1960–009) was used to get a more homogenous film than obtained with a Büchner funnel. The film was air dried overnight, followed by 5 h under dynamic vacuum at room temperature. Discs of 10 mm diameter were punched out, and the film was separated from the Celgard membrane (if not stated otherwise). These freestanding films served as cathodes and were stored under argon atmosphere before cell assembly. The loading of PMTT in the final composite was approximately 0.4 mg cm^−2^. A similar procedure was followed for the PMTT‐CNT film, with the exception of replacing the 50 mL of MXene dispersion with a 50 mL 0.25 g L^−1^ aqueous multi‐walled CNT (Sigma Aldrich, >95 %, 6–9 nm diameter, 5 μm length) dispersion. The CNT dispersion was stabilized by 0.1 wt % cetyltrimethylammonium bromide (CTAB, Sigma Aldrich, >99 %) through 1 h sonication based on another study.^[60]^ The third film, PMTT‐MX‐CNT, was prepared analogously, where 25 mL of the MXene dispersion was mixed with 25 mL of the CNT dispersion by 2 h stirring and 10 min sonication, before dropwise adding the PMTT/acetone solution. Hence, the PMTT content of all films was 50 wt %, and the PMTT loading was approximately 0.4 mg cm^−2^.

Preparation of PMTT‐CB: Reference electrodes were fabricated through drop‐casting a slurry of 50 wt % pristine PMTT, 35 wt % carbon black (Imerys C‐nergy super C65), and 15 wt % polyvinyl alcohol (PVA, Sigma Aldrich, *M*_w_≈61000) binder in water onto stainless steel current collector discs (Goodfellow, 316 L, 25 μm thick). The mixing was done using a mixer mill (Retsch MM400) with a 7 mm stainless steel ball for 45 min at 15 Hz. The electrodes were dried on a hot plate at 60 °C for 30 min, then at room temperature overnight, and lastly 5 h under dynamic vacuum at room temperature. The PMTT loading was approximately 0.2 mg cm^−2^.

**Scheme 2:**
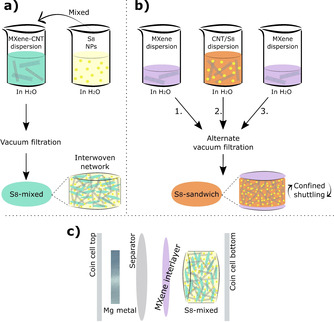
Procedure for preparing (a) S_8_‐mixed and (b) S_8_‐sandwich from Ti_3_C_2_T_*x*_ MXene, CNT, and an aqueous S_8_ nanoparticle dispersion. (c) Schematic principle of using MXene as interlayer with the S_8_‐mixed cathode.

Preparation of S_8_ composites: One of the S_8_ composite films was prepared similar to the PMTT‐MX–CNT composite, but using a S_8_ nanoparticle dispersion [SkySpring Nanomaterials, 99.99 % S_8_ purity, <55 nm, 10 wt % in H_2_O stabilized by polyvinylpyrrolidone (PVP)] instead of the PMTT/acetone solution (illustrated in Scheme 2). In a typical procedure, 0.125 g of the S_8_ dispersion was first diluted with 5 mL deionized H_2_O and sonicated for 15 min. Then, this was added to 50 mL of a 0.25 g L^−1^ MXene‐CNT dispersion (50 : 50 by weight, stabilized by CTAB), to yield a 50 wt% S_8_ composite film after vacuum filtration. A sandwich‐like S_8_ film was also prepared, by first vacuum filtering a certain amount of MXene dispersion (15 mL), followed by a S_8_‐CNT dispersion (30 mL), then a final amount MXene dispersion (10 mL). The 30 mL S_8_‐CNT dispersion was prepared by diluting 0.125 g of the 10 wt % S_8_ nanoparticle dispersion with 5 mL deionized H_2_O, and sonicating this with 25 mL 0.25 g L^−1^ CNT dispersion stabilized by PVP (Aldrich, *M*_w_≈55000) for 1 h. The total composition of the two S_8_ films were thus 50 wt % S_8_, 25 wt % MXene, and 25 wt % CNT, and the S_8_ loading was approximately 0.4 mg cm^−2^. The film with homogeneously mixed constituents is referred to as S_8_‐mixed, whereas the sandwich‐like film is S_8_‐sandwich.

Preparation of MX and MX‐CNT films: To serve as interlayers and/or reference samples, pure MXene and MX‐CNT films without sulfur were obtained by vacuum‐filtering 50 mL of the as‐prepared delaminated MXene dispersion and 50 mL of a MXene‐CNT dispersion (50 : 50 by weight, stabilized by CTAB), respectively. For interlayer purpose, discs of 16 mm diameter were punched out of the prepared film to ensure full coverage of the cathode in the cell. These films also served as references to inspect possible capacity contribution from MXene and MXene–CNT itself. The loading of both films was approximately 0.4 mg cm^−2^.

### Materials characterization

The crystallinity and phase composition of the prepared films were determined by X‐ray diffraction (XRD) using a Bruker D8 Focus Diffractometer, with a CuK_α_ radiation source (*λ*=1.5406 Å) and a 0.2 mm divergence slit. The films were mounted on a monocrystalline Si wafer with the aid of a small amount of vacuum grease. Powder reference samples were dispersed in ethanol or water and distributed onto the Si wafer. The microstructure and elemental composition were inspected by field emission scanning microscopy (LVFESEM, Zeiss SUPRA 55VP or FESEM Zeiss Ultra 55 Limited Edition) equipped with an energy‐dispersive X‐ray spectroscopy (EDS) detector. An acceleration voltage of 2.0 kV was used for microstructure inspection, whereas 10 kV was used for EDS measurements.

### Electrochemical characterization

The prepared films were directly used as cathode in coin cells (Hohsen CR2016, 316 L stainless steel) with a polished Mg metal (Solution Materials) disc as anode and Celgard 2400 as separator. 50 μL of an electrolyte with 0.4 m Mg[B(hfip)_4_]_2_ in dried anhydrous 1,2‐dimethoxyethane (DME, Sigma Aldrich, 99.5 %, inhibitor‐free) was used, prepared as reported earlier.^[20]^ It should be noted that the CTAB‐containing films did not easily detach from the Celgard 3501 membrane used in the vacuum filtration (PMTT‐MX‐CNT, PMTT‐CNT, S_8_‐mixed, MX‐CNT). These films were assembled as cathode including the Celgard 3501, which can be interpreted as an extra separator. Three‐electrode cells (PAT‐cell, EL‐CELL) were assembled similar to the coin cells, but using an electrolyte volume of 110 μL and with a Mg ring reference electrode incorporated into the glass fiber separator. All cells were assembled inside an argon glovebox (MBraun, O_2_<0.1 ppm, H_2_O<0.1 ppm). The coin cells were tested on a Bio‐Logic BCS‐805 cycler and the three‐electrode cells on a Bio‐Logic VMP‐300 potentiostat inside a temperature‐controlled room at 20 °C. Before galvanostatic cycling, a 2 h rest step was conducted to allow the electrolyte to wet the electrodes. The cells were discharged with a current density of 50 mA g^−1^ and charged with a higher current density of 500 mA g^−1^ to reduce polysulfide shuttling, based on a recent study.^[61]^

## Results and Discussion

### PMTT composites

**Figure 1:**
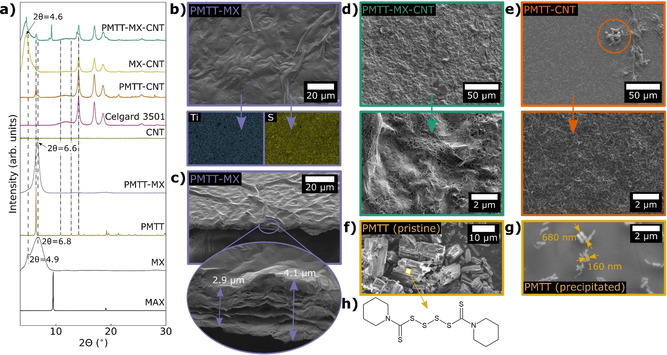
(a) XRD diffractograms of PMTT composite films and reference samples (MX=Ti_3_C_2_T_*x*_, MAX=Ti_3_AlC_2_, Celgard 3501 is a filter membrane/separator). (b) SEM micrograph of PMTT‐MX film with corresponding EDS mapping of Ti and S of the same area. (c) Cross‐section SEM of PMTT‐MX. SEM of (d) PMTT‐MX‐CNT and (e) PMTT‐CNT at low and high magnifications. SEM of (f) pristine PMTT powder and (g) PMTT that was dissolved in acetone and dropwise added into deionized water. (h) Chemical structure of PMTT.

Scheme 1 summarizes the preparation route of the PMTT composites, and Figure 1 shows the XRD diffractograms and SEM micrographs of the obtained films. The removal of the Ti_3_AlC_2_ MAX phase peaks (labelled “MAX” in Figure 1a) and the appearance of a broad peak at low 2*θ* value after etching (labelled “MX” in Figure 1a) confirms the successful selective etching of Al to form Ti_3_C_2_T_*x*_ MXene from LiF and HCl.^[62]^ With the optimized MILD method,^[62]^ intercalated solvated Li‐ions from LiF weaken the hydrogen and van der Waals forces between adjacent sheets, enabling delamination by solely hand shaking. This produces stable colloidal dispersions of delaminated MXenes, which was also observed in this work (Figure S1a in the Supporting Information).

The three prepared PMTT composite films with varying ratio of MXene and CNT displayed different microstructures and crystallinity(Figure 1a–e). The PMTT‐MX film (Figure 1b) resembles the pure MXene film (Figure S1b in the Supporting Information), with a continuous paper‐like morphology and no easily observable PMTT particles. The EDS elemental mapping confirmed a homogeneous distribution of Ti and S (Figure 1b), and cross‐section SEM indicates a film thickness of a few μm (Figure 1c). The XRD of PMTT‐MX revealed no crystalline PMTT, in contrast to the pristine PMTT powder (Figure 1a).

As described in the Experimental Section, the PMTT was dissolved in acetone and added dropwise to the MXene dispersion. To shed light on the process, PMTT was similarly dissolved in acetone and added to deionized H_2_O, forming a stable cloudy white dispersion (Figure S2b in the Supporting Information). Figure 1g shows that the procedure dissolves the several μm‐sized PMTT particles (Figure 1f) and results in considerably smaller rod‐like particles with a diameter of 80–250 nm. Due to the absence of peaks attributable to PMTT in the XRD pattern, it is considered to be amorphous and well dispersed in the PMTT‐MX composite.

The PMTT‐MX–CNT composite displayed a rougher surface, and the high magnification SEM micrograph reveals a homogeneous distribution of MXene flakes in a CNT matrix (Figure 1d). In contrast to the PMTT‐MX film, the XRD showed small crystalline PMTT peaks. This was also seen for PMTT‐CNT, where some PMTT particles could be observed in the SEM (orange circle in Figure 1e). In the preparation process, both PMTT‐MX–CNT and PMTT‐CNT utilized the surfactant CTAB to stabilize the CNT dispersion. Surfactants are known to strongly influence particle formation through nucleation, growth, coagulation, and flocculation.[^64^, ^65^] Hence, CTAB may form micelles or function as a directing agent, offering an explanation to the observation of crystalline PMTT. The characteristic MXene peak was also noteworthy shifted to lower angles for the PMTT‐MX–CNT compared to the pristine MXene (2*θ*=4.6° vs. the bimodal 4.9°/6.8° peak), which was also seen for the MX–CNT reference film. This is attributed to intercalation of CTA^+^, as reported before.^[66]^ It should also be noted that a peak at 2*θ*=9.2° is observed for PMTT‐MX–CNT, attributable to residues of the Ti_3_AlC_2_ MAX phase.

**Figure 2:**
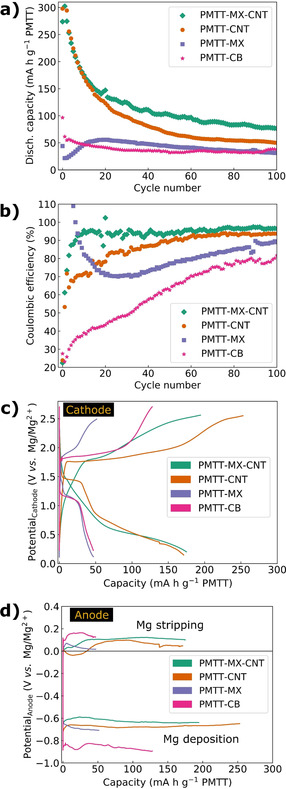
(a) Cycling stability, (b) coulombic efficiency, (c) voltage profiles for the 10th cycle showing the cathode potential, and (d) anode potential for the three PMTT composite films, as well as a PMTT cathode fabricated through conventional casting on stainless steel current collector with carbon black as conductive additive (PMTT‐CB). The PMTT composite films were tested from 0.1 to 3.2 V, the PMTT‐CB to 3.6 V (to avoid premature charge cut‐off in initial cycles), discharging with 50 mA g^−1^ and charging with 500 mA g^−1^.

The galvanostatic cycling of the three PMTT composites is shown in Figure 2, where a PMTT cathode fabricated through conventional casting on a steel current collector with carbon black as conductive additive (referred to as PMTT‐CB) is included for comparison. The PMTT‐MX and PMTT‐CB obtained roughly the same stable discharge capacity of approximately 50 mA h g^−1^ after ten cycles, which is far from the theoretical capacity of 418 mA h g^−1^ PMTT (assuming a 6‐electron reaction). The gradual increase in capacity of the PMTT‐MX composite during the initial cycles resembles the observation by NuLi et al. for another organosulfur compound, which they ascribed to increased active material utilization.^[67]^ This might explain the abnormal coulombic efficiencies above 100 % in the first cycles. In contrast, the PMTT‐MX–CNT and PMTT‐CNT films have an initial discharge capacity of 300 mA h g^−1^. Moreover, the coulombic efficiencies of PMTT‐MX‐CNT and PMTT‐CNT are in general higher than those of PMTT‐MX and PMTT‐CB (Figure 2b), as well as showing a lower overpotential on the cathode (Figure 2c) and anode (Figure 2d). The limited capacity and hence low PMTT utilization for the PMTT‐CB can be explained by the relatively low surface area of carbon black (≈60 m^2^ g^−1^) and the micron‐sized PMTT particles. For PMTT‐MX, the low PMTT utilization is likely influenced by the limited surface area of MXene due to restacking causing a compact structure, as indicated by SEM (Figure 1c). The beneficial role of CNTs to create a more porous MXene composite structure has indeed been reported for Li‐S batteries,^[31]^ explaining the higher capacity of PMTT‐MX‐CNT.

As CTAB‐preintercalated Ti_3_C_2_T_*x*_ has been reported to enable a stable capacity of approximately 100 mA h g^−1^,^[66]^ reference samples of MX and MX‐CNT films without PMTT were similarly tested (Figure S3 in the Supporting Information). Pure MX film demonstrated negligible capacity, and the MX‐CNT a reversible capacity of 25 mA h g^−1^. Consistent with our recent paper on Mg^2+^ intercalation in MXenes,^[68]^ the modest capacity of MX‐CNT should be predominantly attributable to pseudocapacitive surface reactions and proves that PMTT dominates the redox activity in the PMTT composites.

Interestingly, the PMTT‐MX‐CNT exhibited both better capacity retention and higher coulombic efficiency than the PMTT‐CNT film. The improved performance is ascribed to 1) the polar termination groups of MXene, reducing the polysulfide shuttling, and 2) the homogeneous and synergistic mixture of MXene and CNT preventing MXene restacking and forming a porous 3D conductive network. A similar observation was observed for Li‐S batteries by Bao et al.,^[32]^ who reported larger capacities and higher capacity retention for a Ti_3_C_2_T_*x*_/reduced graphene oxide/sulfur composite cathode compared to a solely reduced graphene oxide/sulfur cathode.^[32]^ An analogous beneficial role of MXenes on the cycling stability of Mg‐S batteries is thus confirmed. Still, the somewhat less pronounced discharge and charge plateaus for PMTT‐MX‐CNT compared to PMTT‐CNT are not clear.

The advantage of a current collector‐ and binder‐free cathode architecture is clearly seen when reporting the capacity per mass electrode, that is, including the MXene and/or CNT for the PMTT films, and the carbon black, PVA binder, and stainless‐steel current collector for the PMTT‐CB (Figure S4a in the Supporting Information). Even when the active material loading of a hypothetical high‐energy Mg‐S electrode is increased towards commercial levels, the fraction of inactive mass is substantial (Figure S4b in the Supporting Information). For example, at a high active material loading of 7.5 mg cm^−2^, 49 wt % is inactive mass (current collector, sulfur host material, binder, conductive additive). By using the sulfur host (MXene) itself to form a freestanding film, either a reduced fraction of inactive mass can be achieved (current collector and binder is omitted) or an increased functionality can be enabled (replacing the passive current collector with a material that increase cycling life). As the current collector not only distribute electrons, but also dissipates heat, it is important to note that the thermal conductivity of Ti_3_C_2_T_*x*_ has been reported as 55.8 W m^−1^ K^−1^,^[69]^ which is higher than, for example, stainless steel of 15–40 W m^−1^ K^−1^.[^69^, ^70^]

Despite the improved performance of the PMTT‐MX–CNT film, a rather fast capacity fading is not prevented, and sulfur is observed on the Mg anode after cycling (Figure S5 in the Supporting Information), indicating that the polysulfide shuttling is still largely present. Moreover, the capacity and average voltage are not satisfactory for energy‐dense batteries. As a result, we further developed the synthesis procedure by replacing the PMTT solution with a S_8_ nanoparticle dispersion and optimized the cathode architecture with MXene as a polysulfide‐scavenging interlayer.

### S_8_ composites and MXene interlayer

**Figure 3:**
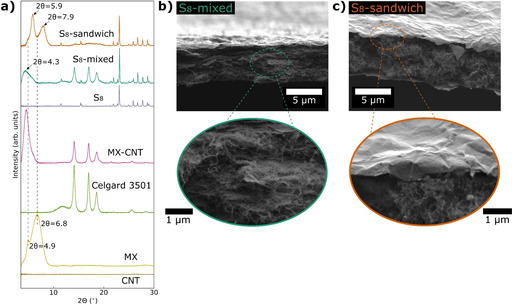
(a) XRD patterns of S_8_ composite films and reference samples (MX=Ti_3_C_2_T_*x*_, Celgard 3501 is a filter membrane/separator) and SEM micrographs of the (b) S_8_‐mixed and (c) S_8_‐sandwich film.

Scheme 2 illustrates the preparation route for the composites based on S_8_ nanoparticles, and the films’ crystallinity and microstructure are depicted in Figure 3. Mixing the S_8_ nanoparticle dispersion with a MXene‐CNT dispersion followed by vacuum‐assisted filtration produced a homogeneous film (Figure 3b, referred to as S_8_‐mixed) where the MXene flakes and CNTs form an interwoven 3D network. The S_8_ nanoparticles are not easily distinguishable by SEM, but their crystalline appearance was verified by XRD (Figure 3a). The characteristic MXene peak was similarly shifted to lower 2*θ* values as observed for the PMTT‐MX‐CNT and MX‐CNT film, consistent with CTA^+^ intercalation from the CTAB surfactant (Figure 3a).

Inspired by reports on MXene as an interlayer for Li‐S batteries,[^33^, ^36^, ^43^, ^48^, ^53^, ^54^, ^55^, ^56^, ^57^, ^58^, ^59^] a more sophisticated S_8_ film was also prepared. The aim was a sandwich‐like architecture, where a network of CNT and S_8_ nanoparticles is sandwiched by two layers of MXene (S_8_‐sandwich in Scheme 2). The lower MXene layer can act as an extremely thin and flexible current collector, the upper layer can serve as an interlayer to confine the polysulfide reservoir between the MXene layers, while the CNTs can supplement the structure with a high surface area for fast kinetics. By simple alternate filtration of a MXene dispersion, a CNT‐S_8_ nanoparticle dispersion and additional MXene dispersion, the sandwich‐like structure was successfullyobtained (Figure 3c). Analogous to the S_8_‐mixed sample, orthorhombic S_8_ was verified by XRD (Figure 3a) after the preparation process. A bimodal characteristic MXene peak was observed for the S_8_‐sandwich film, with slightly higher 2*θ* values than the pure MXene peak (5.9 and 7.9° vs. 4.9 and 6.8°). MXenes are well known to intercalate a range of polar solvents and ions.^[29]^ A possible reason for shifts in the MXene peak positions is thus different amounts of intercalated H_2_O/Li‐ions/surfactants. The different height of the upper and lower MXene layer can also give rise to shift in 2*θ* values.^[71]^

**Figure 4:**
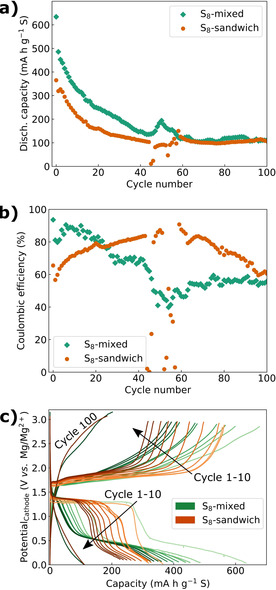
(a) Cycling stability, (b) coulombic efficiency, and (c) voltage profiles of cycle 1–10 for S_8_‐mixed and S_8_‐sandwich films, discharged with 50 mA g^−1^ and charged with 500 mA g^−1^ between 0.1–3.6 V. Note that both cells are three‐electrode cells, where the voltage profiles in (c) show potential of the cathode vs. a Mg reference.

As seen in Figure 4, the S_8_‐mixed demonstrated generally higher discharge capacities than the S_8_‐sandwich film, in addition to higher coulombic efficiencies in the first 25 cycles. However, the voltage profiles (Figure 4c and Figure S6 in the Supporting Information) reveal a higher average discharge voltage for the S_8_‐sandwich. In the first cycle, both films exhibit an equally long first discharge plateau at approximately 1.3 V reaching 270 mA h g^−1^. The S_8_‐mixed film has a distinct second discharge plateau starting at approximately 0.5 V, which is barely seen for the S_8_‐sandwich film. Importantly, from the second cycle, the length of the first discharge plateau at approximately 1.3 V is drastically shortened for the S_8_‐mixed film, whereas the S_8_‐sandwich film maintains the plateau to a larger degree resulting in a higher specific energy of the S_8_‐sandwich for cycle 2–10 (Figure S6 in the Supporting Information). Both films display voltage profiles similar in shape as previously reported Mg‐S batteries with the same electrolyte (note that the voltages in Figure 4 are cathode voltages vs. a Mg reference).[^20^, ^22^, ^72^] In the 100th cycle, no clear voltage plateaus are seen for both films.

Both S_8_‐films demonstrate higher capacities than the PMTT composites (Figure 2), which is not surprising given the much higher theoretical capacity of 1672 vs. 418 mA h g^−1^. The voltage profile of the S_8_‐mixed after cycle two resembles the shape of the PMTT‐MX‐CNT and PMTT‐CNT composites, suggesting similar electrochemical processes that are consistent with earlier observations by our group.^[61]^ The sulfur utilization for the S_8_ composites is limited, in particular for the S_8_‐sandwich, with initial capacities of 635 mA h g^−1^ for S_8_‐mixed and 365 mA h g^−1^ for S_8_‐sandwich. To evaluate the sulfur utilization of the cathode architectures, Li reference cells with a Li metal anode were assembled. As the Li reference cells showed capacities much closer to the theoretical capacity (1300 mA h g^−1^ for S_8_‐mixed, 1480 mA h g^−1^ for S_8_‐sandwich, shown in Figure S7 in the Supporting Information), the limited capacity for the Mg‐S cells seems to be heavily influenced by the Mg‐specific electrochemistry, such as the known issues related to particularly sluggish reaction kinetics,[^16^, ^73^] more evident polysulfide shuttling,^[27]^ and self‐discharge.^[72]^

The relatively absence of the second discharge plateau for the S_8_‐sandwich film in Figure 4c should be elaborated. Using the same electrolyte composition as this work, Häcker et al. showed that the second discharge plateau (corresponding to the liquid–solid conversion of MgS_4_ to MgS) could exhibit as large overpotentials as 1 V at low to moderate temperatures, attributed to slow nucleation of MgS.^[72]^ The sandwich structure offers less available surface area, as the individual MXene sheets are rather densely packed in the upper and lower layer (Figure 3c) and not separated by CNTs as in the S_8_‐mixed film (Figure 3b). The limited surface area appears to notprovide sufficient nucleation sites and can thus explain a large overpotential on the second discharge plateau, causing the cell to prematurely reach the voltage cut off. Still, and somewhat counter‐intuitively, the S_8_‐sandwich maintains the first discharge plateau to a larger degree than the S_8_‐mixed, which is addressed in the following paragraph.

The fact that the S_8_‐sandwich film maintains a higher average discharge voltage than the S_8_‐mixed is intriguing. In a recent study also using the Mg[B(hfip)_4_]_2_ electrolyte, a shortened first discharge plateau (corresponding to the reduction of S_8_ to form MgS_*x*_, where 4≤*x*≤8) was explained by a higher self‐discharge, referring to the dissolution of S_8_ from the cathode followed by a non‐faradaic reduction of S_8_ to MgS_8_ and MgS_6_ at the anode surface.^[72]^ Hence, it appears that the S_8_‐mixed film suffers from serious self‐discharge during the relatively slow discharge of 50 mA g^−1^, which is improved in the sandwich structure. We hypothesized that the reason was the upper layer of MXene in the sandwich structure that could physically and chemically confine the sulfur species and prevent self‐discharge. To verify this, a pure MXene film (1–2 μm thick) was placed on top of the S_8_‐mixed film as an interlayer, discussed below. Moreover, the lower cut‐off voltage was increased to 0.5 V instead of 0.1 V. This has been shown to improve cycling stability, as the poor reversibility of Mg_3_S_8_ and MgS that forms in the end of discharge can be another important reason for capacity fading.^[16]^

**Figure 5:**
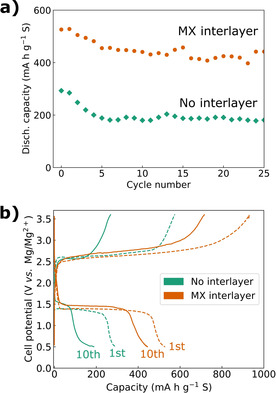
(a) Cycling stability and (b) voltage profiles for the 1st and 10th cycle of the S_8_‐mixed film with and without a Ti_3_C_2_T_*x*_ MXene interlayer between the cathode and the separator. The coin cells were tested between 0.5–3.6 V, discharging with 50 mA g^−1^ and charging with 500 mA g^−1^.

Remarkably, adding the MXene interlayer between the S8‐mixed cathode and the separator (schematically shown in Scheme 2c) roughly doubled the discharge capacity (Figure 5). Moreover, the extra capacity is delivered at the same or higher discharge voltage (≈1.4 V), strongly suggesting a reduced self‐discharge as explained above. Note that there is no apparent increase in overpotential compared to Figure 4c, as the cell potential in a two‐electrode cell is plotted in Figure 5b (the cell potential of the cells in Figure 4c is shown in Figure S6 in the Supporting Information). In stark contrast to the rapid capacity fading presented earlier, the cell with MXene interlayer demonstrated stable cycling with a capacity retention of 83 % after 25 cycles. However, it should be noted that the cell became unstable around cycle 30–40, with severe overcharging (Figure S8 in the Supporting Information). While the [B(hfip)4]‐ anion in the electrolyte may provide a relatively stable solid‐electrolyte‐interphase (SEI) and mitigate polysulfide shuttling,[^72^, ^74^] a recent report has indicated that the SEI is not fully stable for long‐term cycling.^[74]^ Hence, the severe overcharging may be explained by an eventual collapse of the SEI, followed by extensive polysulfide shuttling, and the process is likely accelerated due to the high sulfur utilization with the MXene interlayer. Using Li[B(hfip)4] as electrolyte salt can increase the stability of the SEI while still allowing Mg‐ion conduction.^[74]^ An additional important aspect for future studies is whether the (solvated) Mg‐ions will be transported sufficiently fast through the MXene interlayer at practical electrolyte amounts and sulfur loading. Still, the use of MXene as a cathode constituent and in particular as an interlayer stands out as highly interesting routes for reducing self‐discharge and increasing both the capacity and average discharge voltage for Mg‐S batteries. Given the large group of MXenes and their chemical tunability, an enormous research space is open.

## Conclusions

Several current collector‐ and binder‐free cathode architectures where MXenes play a vital role have been reported. A synergistic effect of MXene and carbon nanotubes (CNTs) was verified, where CNTs provide a high surface area and prevent MXene restacking, while MXene's polar surface groups can reduce polysulfide shuttling and both improve coulombic efficiency and capacity retention. In particular, a MXene interlayer was found to reduce self‐discharge and extend the first discharge plateau, enabling a near doubling of the obtainable discharge capacity (530 vs. 290 mA h g^−1^). Relatively stable cycling with a capacity retention of 83 % after 25 cycles was reported with a MXene interlayer. All in all, MXenes have been added to the list of promising candidates as sulfur host materials for long‐cycle‐life Mg‐S batteries.

## Conflict of interest

The authors declare no conflict of interest.

## Supporting information

As a service to our authors and readers, this journal provides supporting information supplied by the authors. Such materials are peer reviewed and may be re‐organized for online delivery, but are not copy‐edited or typeset. Technical support issues arising from supporting information (other than missing files) should be addressed to the authors.

Supplementary
