# Supplementary material for: Performance Study of MXene/Carbon Nanotube Composites for Current Collector‐ and Binder‐Free Mg–S Batteries
Source: ChemSusChem. 2021 Feb 24;14(8):1864–73. doi: 10.1002/cssc.202100173 (PMC8248395; doi:10.1002/cssc.202100173)
Supplement: Supplementary file 1 — Supplementary [file CSSC-14-1864-s001.pdf]

ChemSusChem

Supporting Information

Performance Study of MXene/Carbon Nanotube Composites for Current Collector- and Binder-Free Mg–S Batteries

Henning Kaland, Frode Håskjold Fagerli, Jacob Hadler-Jacobsen, Zhirong Zhao-Karger, Maximilian Fichtner, Kjell Wiik, and Nils P. Wagner*© 2021 The Authors. ChemSusChem published by Wiley-VCH GmbH. This is an open access article under the terms of the Creative Commons Attribution License, which permits use, distribution and reproduction in any medium, provided the original work is properly cited.

Supplementary data and figures

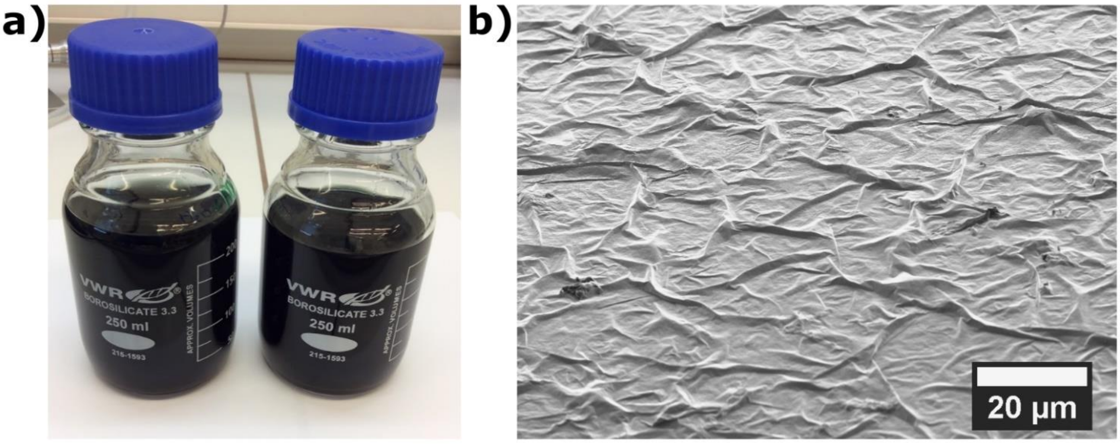

Figure S1. Delaminated $Ti_3C_2T_x$ MXene dispersion in H_2O (a) and SEM of a $Ti_3C_2T_x$ MXene film obtained from vacuum-assisted filtration.

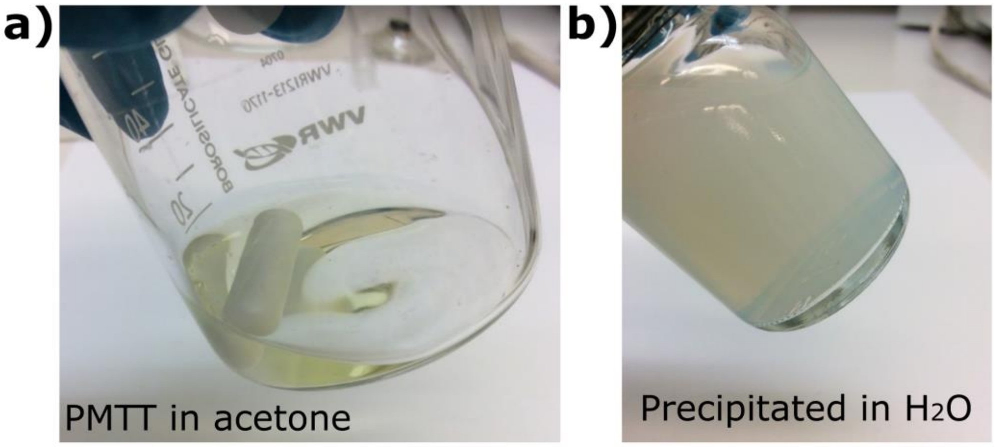

Figure S2. PMTT dissolved in acetone (a) and the resulting dispersion after being dropwise added to H_2O (b).

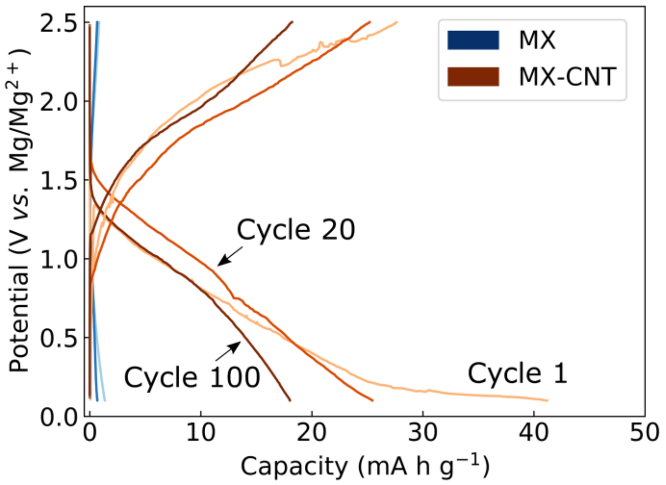

Figure S3. Voltage profiles of cycle 1, 20, 100 for pure $Ti_3C_2T_x$ MXene ("MX" in figure) and $Ti_3C_2T_x$ MXene-carbon nanotube composite ("MX-CNT") without sulfur. Cycled with 50 mA g^{-1} between 0.1-2.5 V.

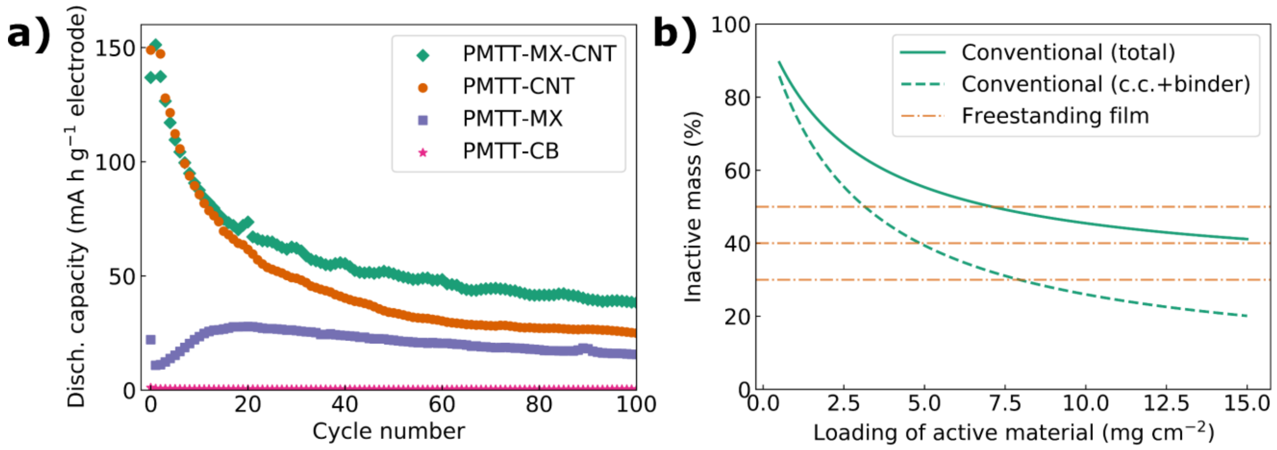

Figure S4. Cycling stability of PMTT composites reported per mass electrode (a), *i.e.* including the MXene and/or CNT for the PMTT films, and the carbon black, PVA binder and stainless steel current collector for the PMTT-CB. Fraction of inactive mass for a conventional electrode and a freestanding film as a function of active material loading (b). The conventional Mg-S electrode (cyan, solid lines) is calculated with 70 wt.% sulfur, 20 wt.% sulfur host material, 5 wt.% binder, 5 wt.% conductive additive, casted on a 15 µm thick Al foil current collector (c.c.), where the inactive mass refers to the sulfur host material, binder, conductive additive and current collector. The cyan dashed lines show the sole contribution of the binder and current collector on inactive mass for the same electrode, which is omitted in freestanding films. The fraction of inactive mass for *e.g.* a MXene-sulfur freestanding film is constant, only determined by the chosen ratio (orange dashed lines show examples of 30, 40 and 50 wt.% MXene).

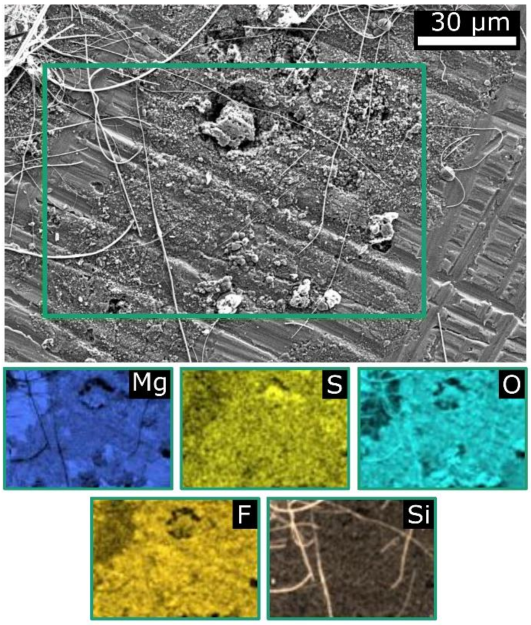

Figure S5. *Post mortem* SEM of the Mg anode from a cycled PMTT-MX-CNT cell, with corresponding EDS mapping. The Si originates from the glass fibre separator.

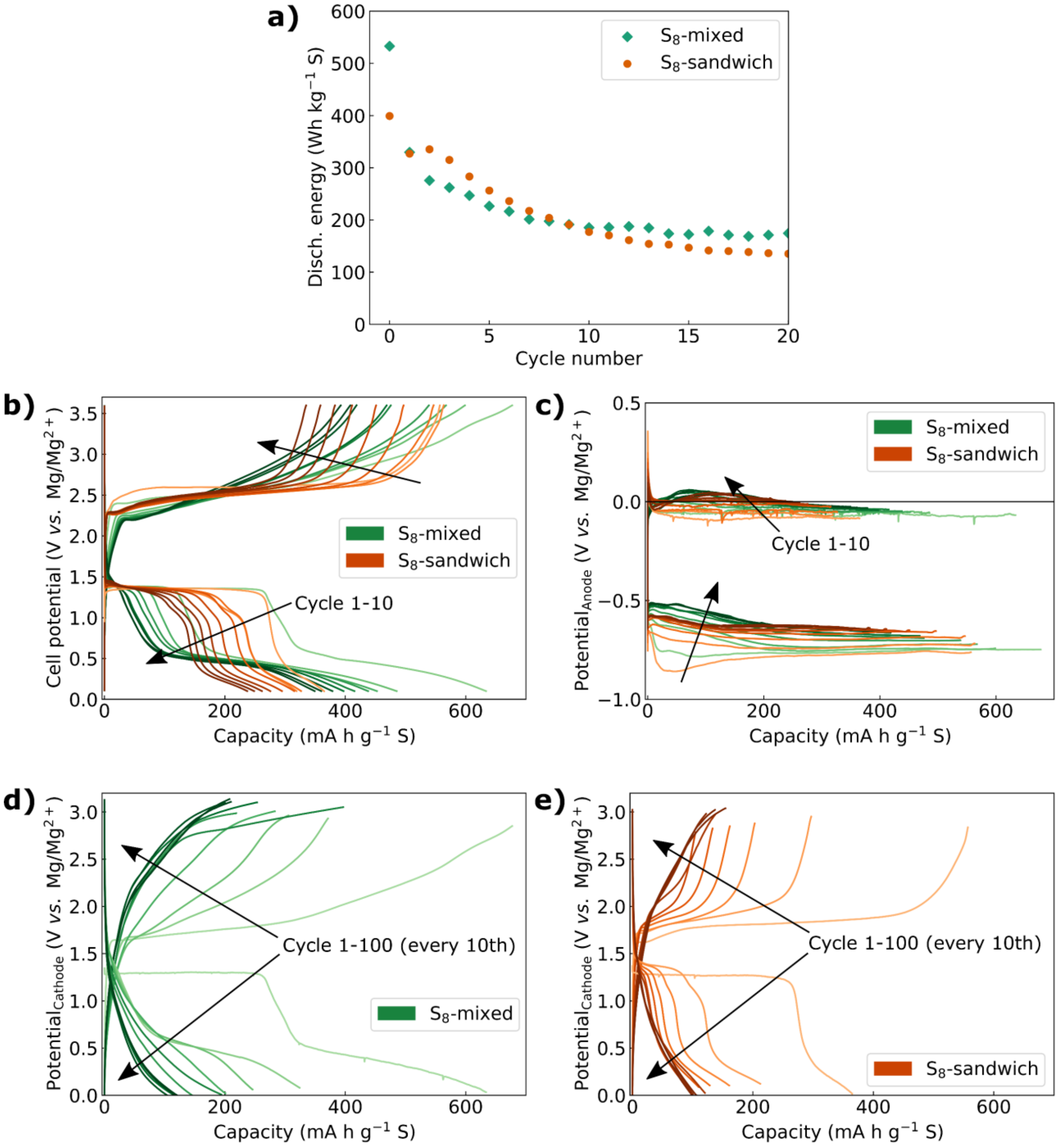

Figure S6. Specific discharge energy per mass sulfur for the S_8 composite films (a), voltage profiles showing cell potential (b), anode potential *vs.* the Mg reference (c) and cathode potential *vs.* the Mg reference for cycle 1, 10, 20, …, 100 for S_8-mixed (d) and S_8-sandwich (e) complementary to the cells presented in Figure 4 in the manuscript.

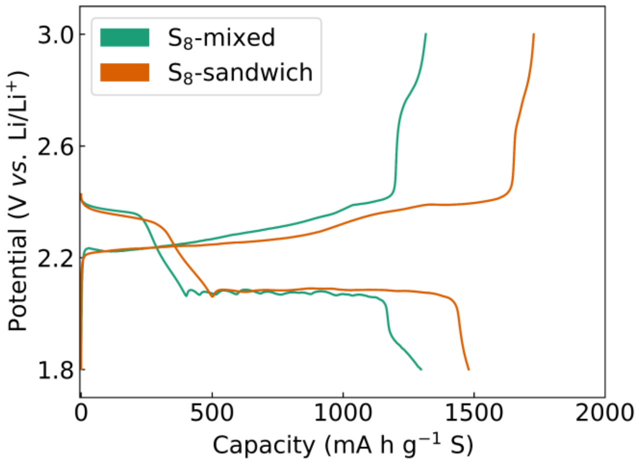

Figure S7. Voltage profiles of Li reference cells (*vs.* Li metal anode) of the S_8 composites, showing the first cycle. An electrolyte of 1 M LiTFSI, 0.1 M LiNO3 in 1,3-Dioxolane/1,2-Dimethoxyethane (v/v 1:1) was used, and the cells were discharged and charged with a current density of 50 mA g^{-1}.

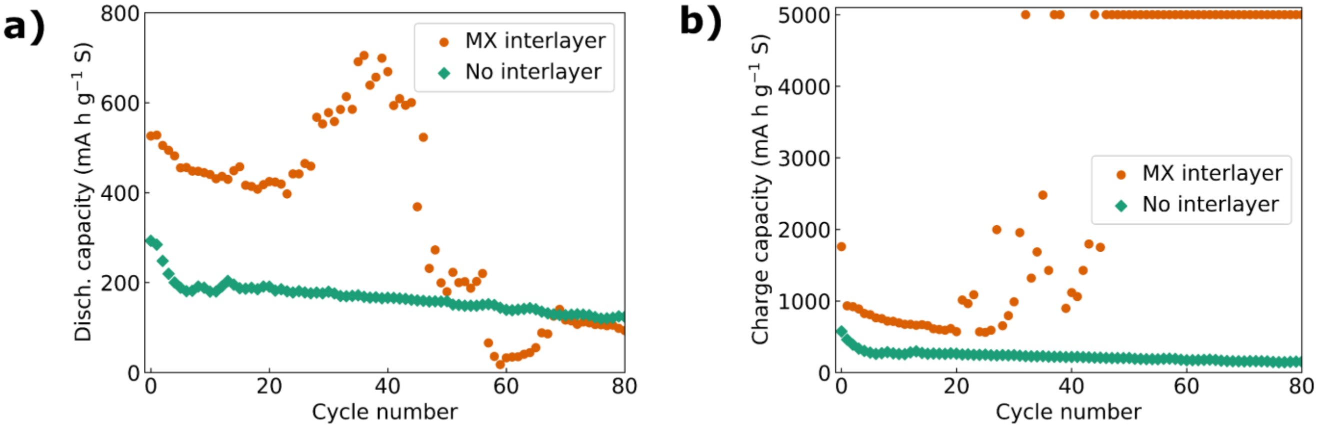

Figure S8. Extended cycling of the S_8-mixed film with and without a $Ti_3C_2T_x$ MXene interlayer, showing specific discharge (a) and charge (b) capacity. The cells were cycled between 0.5-3.6 V, discharging with 50 mA g^{-1} and charging with 500 mA g^{-1}.
